# Iron Depletion in Systemic and Muscle Compartments Defines a Specific Phenotype of Severe COPD in Female and Male Patients: Implications in Exercise Tolerance

**DOI:** 10.3390/nu14193929

**Published:** 2022-09-22

**Authors:** Maria Pérez-Peiró, Mariela Alvarado, Clara Martín-Ontiyuelo, Xavier Duran, Diego A. Rodríguez-Chiaradía, Esther Barreiro

**Affiliations:** 1 Muscle Wasting and Cachexia in Chronic Respiratory Diseases and Lung Cancer Research Group, Pulmonology Department, Department of Medicine and Life Sciences (MELIS), Hospital del Mar, Medical Research Institute (IMIM), Parc de Salut Mar, Universitat Pompeu Fabra (UPF), Barcelona Biomedical Research Park (PRBB), 08003 Barcelona, Spain; 2 Centro de Investigación en Red de Enfermedades Respiratorias (CIBERES), Instituto de Salud Carlos III (ISCIII), 08003 Barcelona, Spain; 3 Pulmonology Department, Hospital Universitario Sant Joan de Reus, 43204 Reus, Spain; 4 Department of Pulmonary Medicine, Hospital Clínic, Institut d’Investigacions Biomèdiques August Pi i Sunyer (IDIBAPS), University of Barcelona, 08036 Barcelona, Spain; 5 Scientific, Statistics, and Technical Department, Hospital del Mar-IMIM, Parc de Salut Mar, 08003 Barcelona, Spain

**Keywords:** severe chronic obstructive pulmonary disease, iron-deficient patients, iron metabolism regulation, pathophysiology of iron regulation, implications in exercise tolerance, systemic manifestations of chronic diseases

## Abstract

We hypothesized that iron content and regulatory factors, which may be involved in exercise tolerance, are differentially expressed in systemic and muscle compartments in iron deficient severe chronic obstructive pulmonary disease (COPD) patients. In the vastus lateralis and blood of severe COPD patients with/without iron depletion, iron content and regulators, exercise capacity, and muscle function were evaluated in 40 severe COPD patients: non-iron deficiency (NID) and iron deficiency (ID) (20 patients/group). In ID compared to NID patients, exercise capacity, muscle iron and ferritin content, serum transferrin saturation, hepcidin-25, and hemojuvelin decreased, while serum transferrin and soluble transferrin receptor and muscle IRP-1 and IRP-2 increased. Among all COPD, a significant positive correlation was detected between FEV_1_ and serum transferrin saturation. In ID patients, significant positive correlations were detected between serum ferritin, hepcidin, and muscle iron content and exercise tolerance and between muscle IRP-2 and serum ferritin and hepcidin levels. In ID severe COPD patients, iron content and its regulators are differentially expressed. A potential crosstalk between systemic and muscle compartments was observed in the ID patients. Lung function and exercise capacity were associated with several markers of iron metabolism regulation. Iron status should be included in the overall assessment of COPD patients given its implications in their exercise performance.

## 1. Introduction

Chronic obstructive pulmonary disease (COPD) is characterized by pulmonary and extrapulmonary manifestations. The extrapulmonary features of COPD include skeletal muscle dysfunction, nutritional abnormalities, cardiovascular alterations, and pulmonary hypertension, among others [1,2]. The systemic component of COPD worsens the patients’ prognosis and their quality of life regardless of the severity of the lung disease [1,2]. Iron deficiency is recognized as an important comorbidity, even in the absence of anemia, in different chronic diseases including chronic heart failure and COPD. It has been estimated that iron deficiency may take place in approximately 40–50% of the COPD patients [3,4,5]. Alterations in iron homeostasis may negatively impact disease progression and functional status in COPD [5,6].

Iron metabolism, which is a tightly regulated process in humans, is an essential component that participates in many physiological processes such as energy metabolism, oxygen transport, and cell growth and differentiation [7,8]. Several molecules and hormones are involved in the regulation of iron release and maintenance of its reservoirs. Processes related to iron transport, uptake, and export to the bloodstream and those involving inflammation are regulated by several molecules such as the iron-sensitive element/iron-sensitive protein (IRE/IRP) system, hepcidin, and hemojuvelin [9,10,11,12,13,14,15,16]. Furthermore, iron content can also be measured in specimens from patients as another marker of iron metabolism [17,18].

In COPD cachectic patients, replicated genes that regulate heme metabolism were downregulated in blood samples [19], while mitochondrial breakdown signaling increased in the vastus lateralis muscle [20]. These findings were directly associated with both disease severity and the loss of mitochondrial content. Serum hepcidin was also shown to be a surrogate biomarker of iron status and metabolism in patients with chronic respiratory diseases including COPD [21]. Iron deficiency in COPD was also associated with reduced physical activity [22]. On the other hand, iron replacement improved exercise capacity and QoL in stable COPD patients with decreased iron content [23]. Studies conducted so far in COPD have focused on the quantification of iron content and regulation in either the blood or the muscle compartment. Assessment of iron metabolism in both muscle and systemic compartments within the same COPD patients is needed. Additionally, comparisons between patients with and without iron deficiency are also to be thoroughly analyzed in order to define a specific phenotypic profile of COPD patients. Moreover, potential associations between iron regulatory factors and exercise tolerance in patients with COPD, with a special emphasis on those with iron deficiency, also warrants a thorough analysis. 

Thus, we hypothesized that iron content and regulatory factors are differentially expressed in both systemic and muscle compartments in patients with severe COPD and muscle dysfunction and iron deficiency compared to a cohort of COPD patients with identical disease severity and iron content levels within the normal range. This approach enabled us to define a specific phenotype of COPD patients characterized by significant alterations in iron regulation in both systemic and muscle compartments along with potential implications in their exercise tolerance and lung function status. Therefore, we sought to investigate in blood and muscle specimens from patients with severe COPD and muscle dysfunction with and without iron deficiency the following parameters and biomarkers: (1) lung function and exercise capacity; (2) hemogram, iron content, and regulators in the blood compartment; (3) regulators of iron homeostasis and iron content in the muscle specimens; and (4) potential associations between iron metabolism biomarkers and both exercise capacity and lung function parameters among all the patients, with a particular purpose in those with iron deficiency. 

## 2. Materials and Methods

### 2.1. Study Population

This was a cross-sectional study, in which forty stable COPD patients (16 female patients) were prospectively and consecutively recruited over the years 2018–2021 from the Department of Respiratory Medicine at Hospital del Mar (Barcelona, Spain). COPD patients were further divided into two different groups: non-anemic iron deficiency (N = 20, 8 female patients) and normal iron content (N = 20, 8 female patients). All the participants were diagnosed according to the Global Strategy of Management of COPD patients (GOLD) criteria, in which the following parameters were included: spirometry values, number of exacerbations, and dyspnea score (modified medical research council, mMRC) [24]. Iron-deficient COPD patients (12 males) presented levels of hemoglobin > 12 g/dL in women and >13 g/dL in men, ferritin < 100 ng/mL, or ferritin 100–299 ng/mL with a transferrin saturation < 20% [21,23,25,26]. The study was approved on 17 January 2018 by the local Ethics Committee at Hospital del Mar (CEIm Parc de Salut Mar, registration # 2017/7691/I). 

### 2.2. Exclusion Criteria

The following exclusion criteria were defined for this study: (1) acute exacerbations in the last three months; (2) other chronic respiratory disease or cardiovascular disorders; (3) neurological, metabolic, kidney, chronic liver disease, or uncontrolled psychiatric disorders; (4) known metabolic or neuromuscular myopathies; (5) treatment with drugs known to alter muscle structure and/or function (e.g., oral corticosteroids); (6) obesity (body mass index > 30 kg/m^2^); (7) history of potentially bleeding conditions; and (8) active oncologic disease.

### 2.3. Anthropometric and Lung Function Assessment

Bioelectrical impedance was used to determined body mass index (BMI) and fat-free mass index (FFMI) of all the patients [27,28,29]. Lung function was assessed through spirometry, measuring the first second of forced expiratory volume (FEV_1_) and forced vital capacity (FVC). Reference values were used to evaluate the resulting values [30,31,32].

### 2.4. Exercise Capacity and Muscle Function Assessment

The six-minute walk test (6-MWT) was performed to evaluate exercise capacity in all COPD patients following previous methodologies [33]. The 6-MWT was executed indoors along a flat, straight, 30 m walking course. During the 6-MWT, patients were encouraged every minute. Patients were allowed to stop to rest if needed. The test was resumed as soon as the patients were able to keep walking. The test lasted for six minutes for all the patients independently of whether they had to stop to rest. 

Upper limb strength was evaluated analyzing hand grip strength using a specific hand dynamometer (Jamar 030J1, Chicago, IL, USA). Three consecutive measurements with the dynamometer were obtained from each patient, with a maximum of <5% variability between them. The higher measurement was used as the maximum voluntary contraction of the flexor muscles of the hands. In the analysis, reference values from Luna-Heredia et al. were used [34]. 

Lower limb strength was evaluated assessing quadriceps muscle strength through the determination of isometric maximum voluntary contraction (QMVC) of lower limbs as previously described [35]. For these measurements, a fixed handheld dynamometer (MicroFet 2^TM^, Hoggan Scientific, Salt Lake City, UT, USA) was situated on the anterior tibia of the patient and the QMVC was recorded through the exerted compression force. Three different measurements were obtained for each subject (<5% variability among them), accepting the highest value as the QMVC. In the analysis, reference values from Seymour et al. [36] were used.

### 2.5. Blood Samples and Muscle Biopsies

Blood samples were obtained from the arm vein after an overnight fasting period. To evaluate the iron content in the patients, the following blood parameters were assessed: hemoglobin, hematocrit, mean corpuscular (erythrocyte) volume (MCV), mean corpuscular hemoglobin (MCH), mean corpuscular hemoglobin concentration (MCHC), serum iron, transferrin, transferrin saturation, serum ferritin, and soluble transferrin receptor. In order to determine levels of the serum hepcidin and hemojuvelin, blood was collected into serum tubes with clot activator (Vacuette^®^, Kremsmünster, Austria). Serum tubes with the blood samples were centrifuged at 1600× *g* for 15 min to obtain the serum. Immediately, serum samples were stored at −80 °C for further use.

As previously described, specimens from the vastus lateralis muscle from all study participants were acquired using the open biopsy technique [27,28,29]. Muscle samples were immediately frozen in liquid nitrogen and then stored at −80 °C (temperature controlled with alarm control) for further molecular experiments. Additionally, a remaining specimen of muscle were immersed in an alcohol-formol bath to be thereafter embedded in paraffin. Paraffin-embedded samples were used for the assessment of structural modifications. 

### 2.6. Biological Analyses

*Muscle iron content analysis*. Iron concentration in vastus lateralis specimens were measured using the Iron colorimetric assay kit (Elabscience, Houston, TX, USA) following the manufacturer’s instructions. Vials 1 and 2 were provided by the manufactured kit and were used as recommended by the company. To analyze the iron content in the muscle specimens, 20 mg of frozen tissue was cleaned with PBS to remove traces of blood. Samples were immediately homogenized in PBS (0.01 M. pH 7.4) using a tissue homogenizer. The Bradford assay was applied in order to quantify protein concentration in the samples as previously described [28,29,37]. Briefly, protein quantification was quantified in triplicates for each sample and bovine serum albumin (BSA) (NZYtech, Lisbon, Portugal) was used as the standard. Briefly, the dye reagent concentrate (Bio-Rad Inc., Hercules, CA, USA) reacted with proteins, producing a change in absorbance at 595 nm that could be detected using a spectrophotometer. In order to quantify the iron content, 2 mg/L iron standard working solution was prepared by mixing reagent 1 in deionized water in a volume ratio of 1:49. An iron chromogenic agent was prepared following the kit instructions. Samples were subsequently 2:3 diluted in deionized water. The iron chromogenic agent was added to the tubes containing the samples, the standard, and the blank (500 µL deionized water). Tubes were vortexed for several seconds up until full homogenization was attained. Then, the tubes were incubated at 100 °C in a water bath for 5 min. After cooling down the samples with running water, tubes were centrifuged at 2300× *g* for 10 min. Then, 200 µL of the supernatant of each tube was transferred to the 96-well plates in a plate-reader (Infinite M Nano, Tecan Group Ltd., Zürich, Switzerland), and optical density (OD) values were measured at 520 nm wavelength. Intra-assay coefficients of variation for all the samples ranged from 0.07% to 7.60%. To calculate the iron concentration within the muscle specimens, the following mathematical formula was applied:

$$\mathrm{Vastus}\;\mathrm{lateralis}\;\mathrm{iron}\;\mathrm{content}\;\left(\frac{\mathrm{mg}}{\mathrm{gprot}}\right)=\frac{{\mathrm{OD}}_{\mathrm{sample}\;}-{\;\mathrm{OD}}_{\mathrm{blank}}}{{\mathrm{OD}}_{\mathrm{standard}}-{\;\mathrm{OD}}_{\mathrm{blank}}}\;\times \;2\;\mathrm{mg}/L\;\times \;\mathrm{protein}\;\mathrm{concentration}$$

*Hemojuvelin*. Determination of serum hemojuvelin concentration was assessed using Human HJV (Hemojuvelin) ELISA Kit (Elabscience, Houston, TX, USA) following the manufacturer’s instructions. Briefly, 100 µL 50-fold diluted serum samples and standards were added in the corresponding wells of the hemojuvelin antibody pre-coated 96-well plate. Then, samples were incubated at 37 °C for 90 min. After decanting the liquid, 100 µL of the biotinylated antibody was added to each well and samples were incubated 1 h at 37 °C. Afterwards, samples were washed three times and consecutively 100 µL of HRP conjugate working solution was poured to each well. After an incubation of 30 min at 37 °C, samples were washed five times. Then, 90 µL of substrate reagent was added to each well and samples were incubated 15 min at 37 °C. Following this incubation, stop solution was added to each well to stop the enzyme substrate reaction. Absorbance of each sample was read at 450 nm wavelength. A standard curve was always generated with each assay run. Intra-assay coefficients of variation for all the samples ranged from 0.11% to 6.93%.

*Hepcidin-*25. Determination of serum Hepcidin-25 concentration was assessed using Human hepcidin (Hepc) ELISA kit (Biorbyt, Cambridgeshire, United Kingdom) following the manufacturer’s instructions and previously described methodologies [21]. Briefly, in each well of the hepcidin antibody pre-coated microplate, 50 µL 5-fold diluted serum samples or standard and 50 µL HRP-conjugate were poured. The microplates containing the samples were then incubated at 37 °C for 1 h and, subsequently were washed three times. Successively, 50 µL substrate A and 50 µL substrate B were added and incubated at 37 °C for 15 min. Finally, the enzymatic reaction was stopped by adding 50 µL stop solution. Immediately afterwards, absorbance of each sample was read at 450 nm wavelength. A standard curve was always generated with each assay run. Intra-assay coefficients of variation for all the samples ranged from 0.11% to 10.98%. 

*Immunoblotting.* Frozen muscle samples from vastus lateralis of all study patients were homogenized using a specific lysis buffer containing 50 mM 4-(2-hydroxyethyl)-1-piperazineethanesulfonic acid (HEPES), 150 mM NaCl, 100 nM NaF, 10 mM Na pyrophosphate, 5 mM EDTA, 0.5% Triton-X, 2 μg/mL leupeptin, 100 μg/mL phenylmethylsulfonyl fluoride (PMSF), 2 μg/mL aprotinin, and 10 μg/mL pepstatin A. Protein concentration of each muscle homogenate was determined using the Bradford method as previously described [27,28,29,37]. Between 5–20 micrograms of protein samples (according to antigen and antibody) were diluted 1:1 with 2X laemmli buffer (Bio-Rad Laboratories, Inc., Hercules, CA, USA) with 10% of 2-mercaptoethanol (Bio-Rad Laboratories, Inc., Hercules, CA, USA). Then, samples were boiled at 95 °C for five minutes and proteins were after separated by electrophoresis. Following the electrophoresis, proteins were transferred onto polyvinylidene difluoride (PVDF) membranes (Merck KGaA, Darmstadt, Germany). Prior to primary antibody incubation, membranes were blocked with bovine serum albumin (BSA) (NZYTech) or with 5% nonfat milk. The following primary antibodies were incubated overnight at 4 °C to analyze protein content of desired molecular markers, which include: ferritin (anti-ferritin antibody, Abcam Cambridge, UK), myoglobin (anti-myoglobin antibody, Santa Cruz Biotechnology, Dallas, TX, USA), ferroportin-1 (anti-ferroportin-1 antibody, Santa Cruz Biotechnology), transferrin receptor (anti-transferrin receptor antibody, Santa Cruz Biotechnology), IRP-1 (anti-IRP-1 antibody, Santa Cruz Biotechnology), IRP-2 (anti-IRP-2 antibody, Abcam), and glyceraldehyde-3-phosphate dehydrogenase (GAPDH, anti-GAPDH antibody, Santa Cruz Biotechnology). The following day, PVDF membranes were incubated for 1 h at room temperature with HRP-conjugated secondary antibodies (Jackson ImmunoResearch Inc., West Grove, PA, USA). In all samples, the desired antigens were detected using a chemiluminescence kit (Thermo Scientific, Rockford, IL, USA) and the Alliance Q9 Advanced (Uvitec Cambridge, UK) imager. Membranes from the different groups were jointly revealed under the same exposure conditions. OD from the resulting bands were quantified using the ImageJ software (National Institute of Health, available at http://rsb.info.nih.gov/ij/, accessed on 1 June 2022). Finally, optical density values corresponding on each protein of interest were normalized with optical density values of glycolytic enzyme GAPDH in all the immunoblots. Negative control experiments were conducted in this set of experiments. For this purpose, primary antibodies were omitted for each given marker and membranes were revealed with the corresponding secondary antibody only. 

### 2.7. Statistical Analysis

Results are expressed in tables and graphs as mean (standard deviation). In the graphs, the green color dots or triangles indicate male patients in either study group, while the blue color dots or triangles indicate female patients in the same groups. In order to explore the potential influence of gender in the comparisons of variables between the two study groups, a linear regression analysis was calculated for all the variables (clinical and biological). Potential associations between clinical and biological variables were assessed using the Pearson’s correlation coefficients. Such correlations were explored in all the COPD as a group and within each group of patients individually. Correlations were targeted specifically for variables whose mean values showed a statistically significant difference between the two study groups. Additionally, graphical correlation matrixes were depicted using the R package corrplot (https://cran.r-project.org/web/packages/corrplot/index.html, accessed on 15 June 2022). Blue dots indicated the existence of a positive correlation between two variables, while the red dots represented negative correlations. Additional multivariate linear regression analyses were used to test the associations between ferritin, hepcidin, and muscle iron content with the six-minute walk distance (meters and % predicted), in which age, sex, and FEV_1_ were the adjusted variables. Sample size was calculated using the hepcidin as the target variable. In a two-sided test, accepting an alpha risk of 0.05 and a beta risk of 0.2 (80% power), a minimum of 16 patients in each group were required to detect a difference of at least 200 ng/mL of hepcidin. Statistical significance was established at *p* ≤ 0.05. Actual *p* values are reported in both tables and figures. All the statistical analyses were carried out using the statistical software SPSS 23.0 (SPSS Inc., Chicago, IL, USA).

## 3. Results

### 3.1. General Clinical Features of the Study Patients

As can be seen in Table 1, clinical characteristics (anthropometry, smoking history, lung function, and GOLD classification) of the patients were similar in both study groups. Exercise capacity as measured by the six-minute walk test was significantly reduced in iron deficiency patients when compared to non-iron deficiency patients (Table 2). Nonetheless, no significant differences were detected in either upper or lower limb muscle function between the two groups of patients (Table 2). Iron metabolism parameters are shown in Table 3. Serum levels of ferritin, transferrin saturation, and muscle iron content were lower in the iron deficiency than in non-iron deficiency patients, while those of the soluble transferrin receptor and transferrin were higher in the former patients (Table 3).

### 3.2. Associations between Clinical Parameters and Serum Iron Metabolism Markers

**Figure 1:**
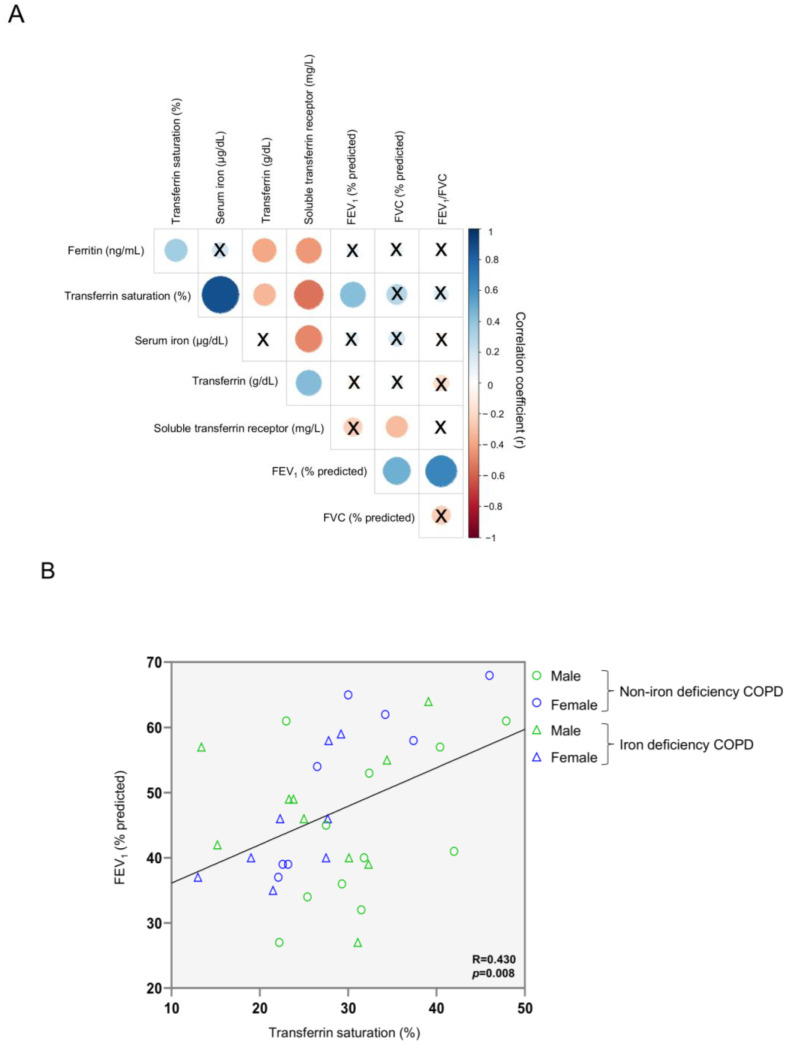
Correlation matrix of blood iron metabolism parameters and lung function parameters in all COPD patients (**A**) and scatter plot representation of correlation between FEV_1_ and transferrin saturation in all COPD patients (**B**). In matrix, blue color represents positive correlations, while negative correlations are indicated in red. Crosses inside the circle mean that the correlation is not statistically significant (*p* value > 0.05). The correlation coefficients (Y axis of the graph) are proportional to the circle color intensity and size. In the scatter plot, the green color indicates male patients, while the blue color indicates female patients. The dots represent the patients with non-iron deficiency while the triangles represent the patients with iron deficiency. Abbreviations: FEV_1_, first second of forced expiration; FVC, forced vital capacity; COPD, chronic obstructive pulmonary disease.

Among all patients, a significant positive correlation was observed between FEV_1_ predicted and transferrin saturation (Figure 1A). Figure 1B shows the distribution of all the patients including both females and males in both groups (blue symbols represent the female while green symbols represented the male patients). Moreover, significant inverse correlations were detected between serum levels of ferritin, transferrin saturation, and serum iron and those of soluble transferrin receptor (Figure 1A and Figure S1A,B, respectively), whereas a positive association was seen between the latter parameter and transferrin levels (Figure 1A and Figure S1C–E, respectively). Furthermore, serum levels of transferrin positively correlated with soluble transferrin receptor, and negatively correlated with serum ferritin and transferrin saturation (Figure 1A and Figure S1F–H, respectively).

**Figure 2:**
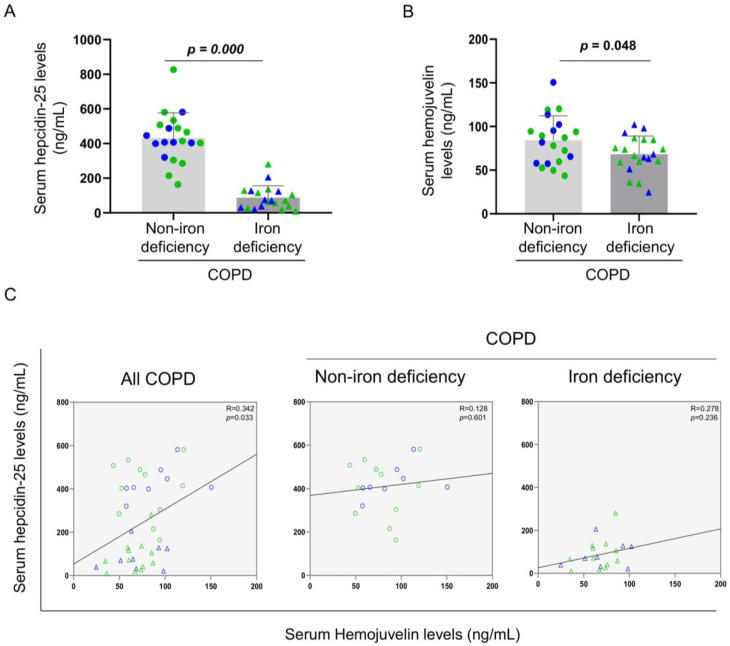
Mean values and standard deviation of (**A**) serum hepcidin-25 (ng/mL) and (**B**) hemojuvelin (ng/mL) variables in COPD patients. (**C**) Scatter plots representation of correlations between serum hepcidin and serum hemojuvelin in all COPD patients (**left** panel), non-iron deficiency (**middle** panel) and iron deficiency (**right** panel) COPD patients. In the graphs, the green color dots or triangles indicate male patients in either study group, while the blue color dots or triangles indicate female patients in the same groups. Definition of abbreviations: COPD, chronic obstructive pulmonary disease. Adjusted *p* values for gender differences are shown in the graphs.

Serum levels of hepdicin-25 and hemojuvelin were significantly reduced in iron deficiency patients when compared to non-iron deficiency patients (Figure 2A,B, respectively). Furthermore, significant positive correlations were observed in these two serum parameters among all COPD patients, but not when analyzed independently (Figure 2C, respectively).

**Figure 3:**
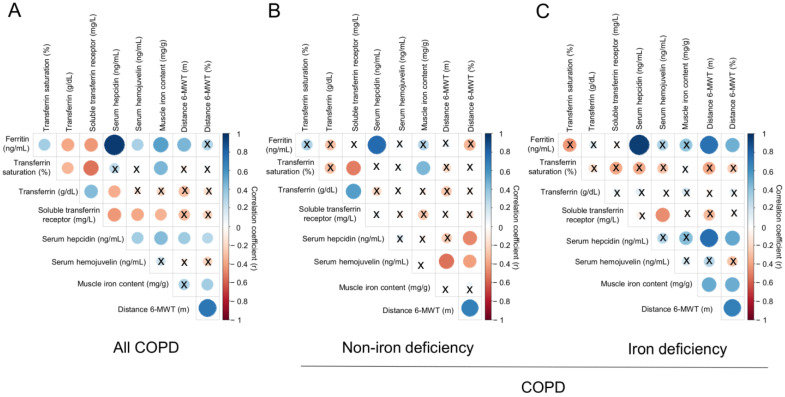
Correlation matrix of the iron metabolism parameters and six-minute walk test distance in (**A**) all COPD patients, (**B**) non-iron deficiency and (**C**) iron deficiency COPD patients. In matrix, blue color represents positive correlations, while negative correlations are indicated in red. Crosses inside the circle mean that the correlation is not statistically significant (*p* value > 0.05). The correlation coefficients (Y axis of the graph) are proportional to the circle color intensity and size. Abbreviations: 6-MWT, six-minute walk test. Definition of abbreviations: COPD, chronic obstructive pulmonary disease.

**Figure 4:**
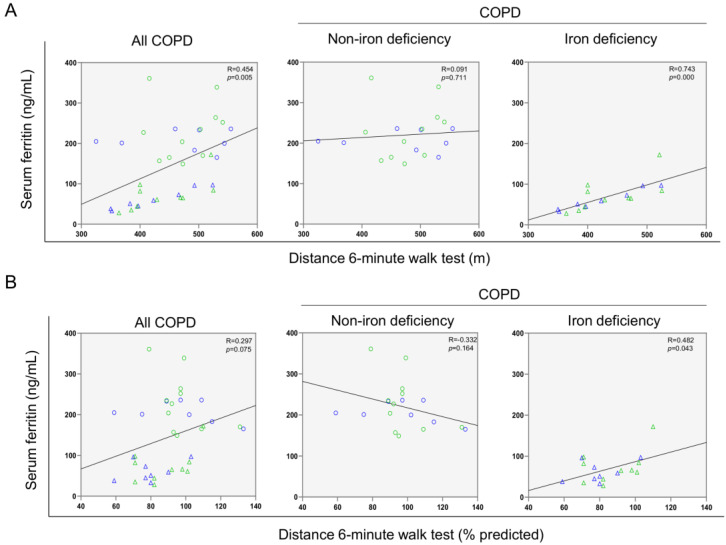
Scatter plots representation of correlations between serum ferritin levels and six-minute walk test distance (m) (**A**) and six-minute walk test distance (% predicted) (**B**) in all COPD patients (**left** panels), non-iron deficiency (**middle** panels) and iron deficiency (**right** panels) COPD patients. In the scatter plots, the green color dots or triangles indicate male patients in either study group, while the blue color dots or triangles indicate female patients in the same groups. Definition of abbreviations: COPD, chronic obstructive pulmonary disease.

**Figure 5:**
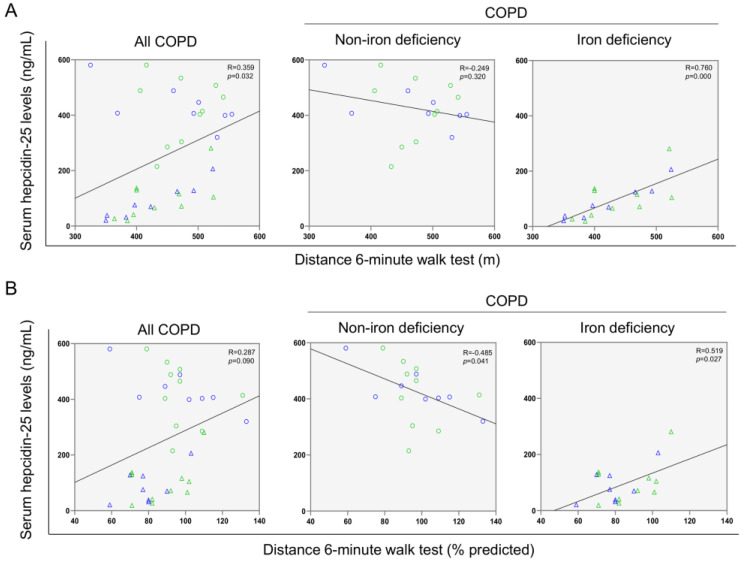
Scatter plots representation of correlations between serum hepcidin levels and distance six-minute walk test (m) (**A**) and distance six-minute walk test (% predicted) (**B**) in all COPD patients (**left** panels), non-iron deficiency (**middle** panels) and iron deficiency (**right** panels) COPD patients. In the scatter plots, the green color dots or triangles indicate male patients in either study group, while the blue color dots or triangles indicate female patients in the same groups. Definition of abbreviations: COPD, chronic obstructive pulmonary disease.

**Figure 6:**
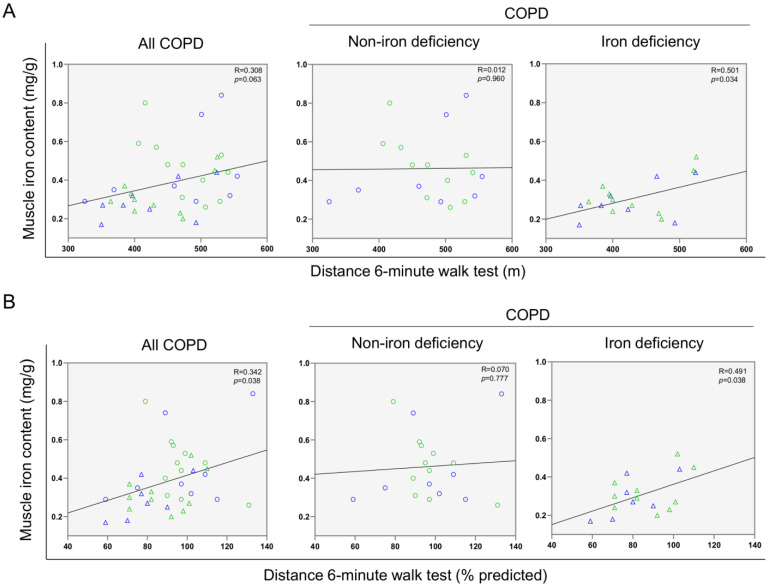
Scatter plots representation of correlations between muscle iron content and distance six-minute walk test (m) (**A**) and distance six-minute walk test (% predicted) (**B**) in all COPD patients (**left** panels), non-iron deficiency (**middle** panels) and iron deficiency (**right** panels) COPD patients. In the scatter plots, the green color dots or triangles indicate male patients in either study group, while the blue color dots or triangles indicate female patients in the same groups. Definition of abbreviations: COPD, chronic obstructive pulmonary disease.

**Figure 7:**
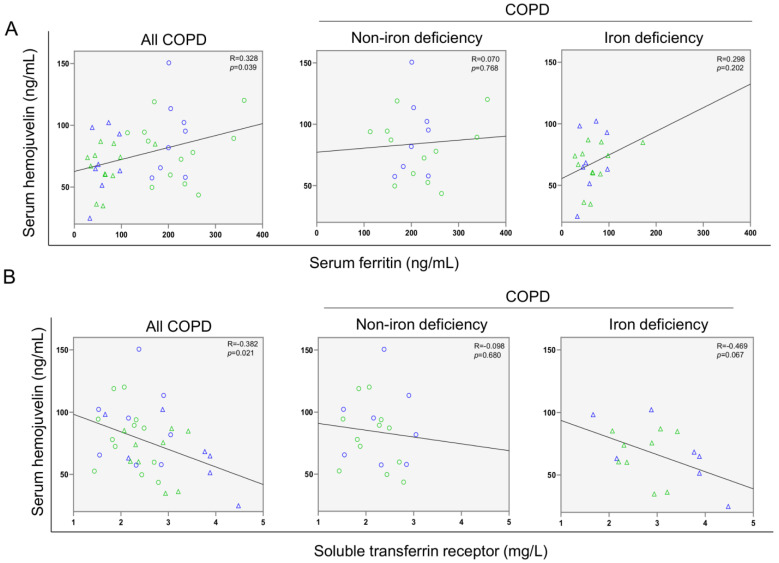
Scatter plots representation of correlations between serum hemojuvelin levels and serum ferritin (**A**) and soluble transferrin receptor (**B**) in all COPD patients (**left** panels), non-iron deficiency (**middle** panels) and iron deficiency (**right** panels) COPD patients. In the scatter plots, the green color dots or triangles indicate male patients in either study group, while the blue color dots or triangles indicate female patients in the same groups. Definition of abbreviations: COPD, chronic obstructive pulmonary disease.

A significant correlation was found between serum ferritin levels and the walked distance in meters when all the patients were analyzed together (Figure 3 and Figure 4A). Such a correlation was lost in non-iron deficiency patients (Figure 3 and Figure 4A), while positive correlations were again detected between serum ferritin levels and the walked distance (meters and predicted variables) among iron deficiency patients. These correlations were maintained after adjusting for age, sex, and FEV_1_ in these patients (Figure 3 and Figure 4A,B, respectively). When all the patients were considered, positive correlations were detected between either serum hepcidin levels or muscle iron content and the walked distance (Figure 3, Figure 4A,B and Figure 5A,B, respectively). In non-iron deficiency patients, however, negative correlations were observed between serum hepcidin levels and the walked distance (predicted variables, Figure 3 and Figure 4A,B, respectively) and between serum hemojuvelin levels and the latter parameter (meters and predicted variables, Figure 3). Additionally, among iron deficiency patients, positive associations were also seen between either serum hepcidin or iron content levels and the walked distance (meters and predicted variables, Figure 3, Figure 5A,B and Figure 6A,B, respectively). These correlations were maintained after adjusting for age, sex, and FEV_1_ in these patients. Adjusted *p* values resulting from the multivariate linear regression showed statistically significant associations between distance 6 min walk test (m) and ferritin (*p* = 0.001), hepcidin (*p* = 0.001), and muscle iron content (*p* = 0.028), and between distance 6 min walk test (% predicted) and ferritin (*p* = 0.023), hepcidin (*p* = 0.025), and muscle iron content (*p* = 0.032) in the iron deficiency group. Among all the COPD patients, serum levels of hemojuvelin were also positively associated with those of ferritin, while they correlated negatively with those of soluble transferrin receptor (Figure 3 and Figure 7A,B, respectively).

### 3.3. Iron Metabolism in the Vastus Lateralis

**Figure 8:**
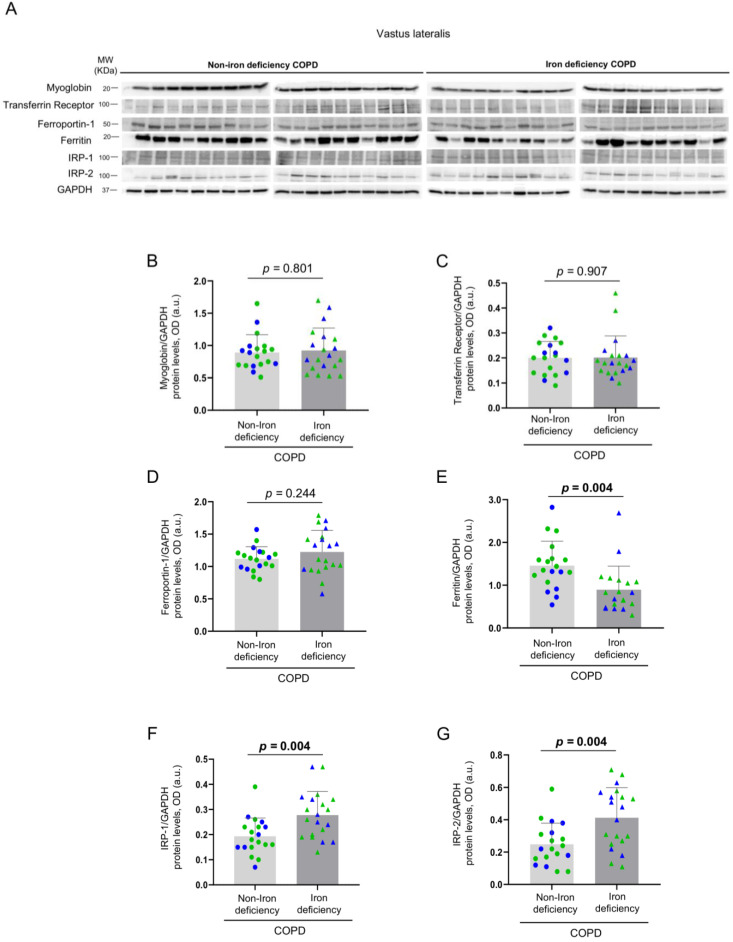
Representative immunoblots of myoglobin, transferrin receptor, ferroportin-1, ferritin, IRP-1, IRP-2 and GAPDH proteins in the vastus lateralis of all COPD patients (**A**). Mean values and standard deviation of myoglobin (**B**), transferrin receptor (**C**), ferroportin-1 (**D**), ferritin (**E**), IRP-1 (**F**) and IRP-2 (**G**) protein content as measured by optical densities in arbitrary units (OD, a.u.). In the graphs, the green color dots or triangles indicate male patients in either study group, while the blue color dots or triangles indicate female patients in the same groups. Definition of abbreviations: IRP, Iron regulatory protein; GAPDH, glyceraldehyde-3-phosphate dehydrogenase; MW, molecular weight; kDa, kilodalton. Adjusted *p* values for gender differences are shown in the graphs.

**Figure 9:**
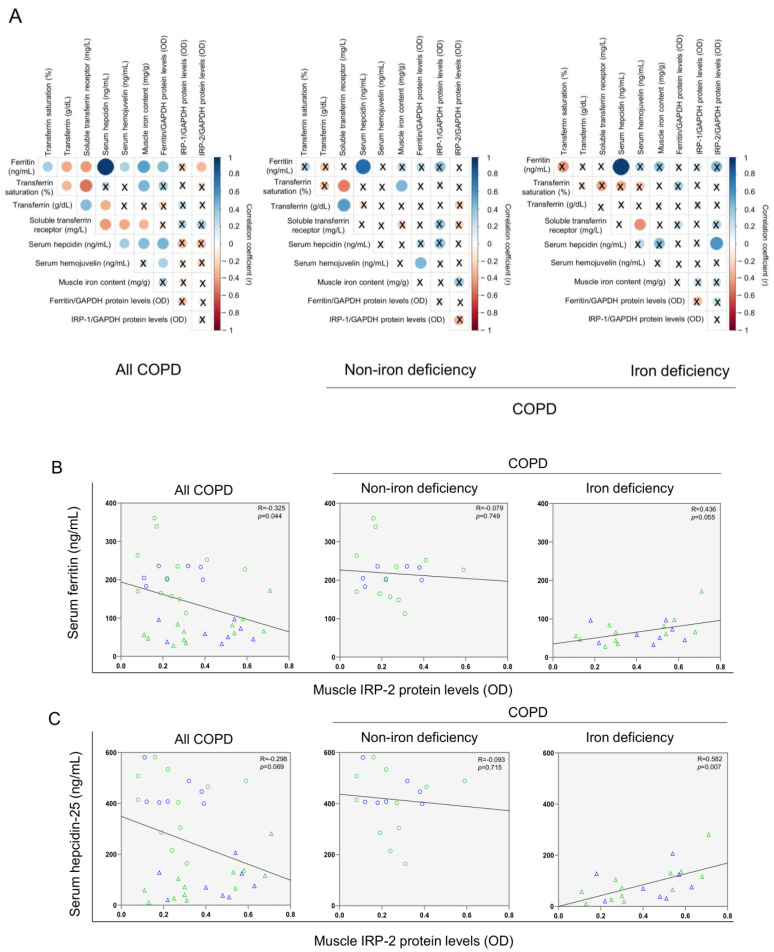
Correlation matrix of the blood and muscle iron metabolism parameters in all COPD patients (**left** panels), non-iron deficiency (**middle** panel) and iron deficiency (**right** panel) COPD patients (**A**). In matrix, blue color represents positive correlations, while negative correlations are indicated in red. Crosses inside the circle mean that the correlation is not statistically significant (*p* value > 0.05). The correlation coefficients (Y axis of the graph) are proportional to the circle color intensity and size. Scatter plots representation of correlations between serum ferritin and muscle IRP-2 protein levels (**B**), and serum hepcidin and muscle IRP-2 protein levels (**C**), in all COPD patients (left panels), non-iron deficiency (middle panel) and iron deficiency (right panel) COPD patients. In the scatter plots, the green color dots or triangles indicate male patients in either study group, while the blue color dots or triangles indicate female patients in the same groups. Definition of abbreviations: IRP, Iron regulatory protein; GAPDH, glyceraldehyde-3-phosphate dehydrogenase; MW, molecular weight.

No significant differences in muscle levels of myoglobin, transferrin receptor, and ferroportin-1 were observed between the two study groups (Figure 8A–D), whereas levels of ferritin were significantly lower in iron deficiency than in non-iron deficiency patients (Figure 8E). Protein content of IRP-1 and IRP-2 increased in the vastus lateralis of iron deficiency compared to non-iron deficiency patients (Figure 8F,G, respectively). Significant negative correlations were observed between either serum ferritin or serum hepcidin-25 levels and those of muscle IRP-2 among all COPD patients, while in iron deficiency patients positive correlations were found between the same variables (Figure 9A–C). In non-iron deficiency patients, no significant correlations were observed between either serum ferritin or serum hepcidin, and muscle IRP-2 content (Figure 9A–C).

## 4. Discussion

The most relevant findings encountered in the investigation are that in severe COPD with iron deficiency, iron content and its regulators are differentially expressed compared to patients with identical degree of disease severity and muscle weakness with normal iron content. For the same degree of the airway obstruction, exercise capacity as measured by the six-minute walking test was reduced in the iron-deficient patients. Moreover, in the latter patients, levels of serum and muscle iron and ferritin content, and serum transferrin saturation, were also lower than in the non-iron deficient patients, whereas the content of transferrin and that of the soluble receptor of transferrin were greater. Airway obstruction as measured by FEV_1_ positively correlated with serum transferrin saturation among all the study patients. Significant positive correlations were also detected between serum ferritin, hepcidin, and muscle iron content and exercise tolerance among the iron-deficient patients. In these patients compared to the non-iron deficient group, serum levels of hepcidin-25 and hemojuvelin decreased, while those of IRP-1 and IRP-2 increased. Positive associations were also detected between serum hepcidin and muscle IRP-2 levels among the patients with iron deficiency. These results remained equal when adjusted by gender. Iron deficiency defined a specific profile of patients with severe COPD and muscle weakness regardless of gender in this study. These are relevant novel results that shed light onto the potential implications of iron content and its regulation in the exercise capacity and the systemic component of patients with severe COPD.

Non-anemic iron deficiency can be encountered in up to 50% of COPD patients [3,4,5]. Iron deficient COPD patients showed a dysregulation of the iron metabolism parameters that were assessed specifically in their systemic compartment. Nonetheless, serum iron levels did not significantly differ between the two groups of COPD patients. As serum iron levels may fluctuate throughout the day, this parameter may not be all that reliable to properly diagnose the iron deficiency in patients [38,39,40]. At initial stages, iron deficiency can be defined as a result of reduced iron storage, which may translate into decreased serum ferritin levels [38,39,40]. Interestingly, serum iron concentration may remain within normal ranges in those early phases of iron depletion. As no significant differences in serum iron levels were observed between the two study groups, it may be possibly concluded that COPD iron deficient patients were probably at their initial stages of the iron depletion process [38,41]. Exercise capacity, however, was indeed reduced in the iron deficient patients. This finding suggests that decreased exercise capacity may be a clinical surrogate of iron depletion in chronic disease such as in COPD.

A positive strong correlation between ferritin levels and the six-minute walk distance was also seen in the iron deficient COPD patients. These results are consistent with previous findings. As such, the existence of a correlation between submaximal exercise capacity and the ferritin index was also detected in patients with chronic heart failure [42]. A positive correlation between the six-minute walk test distance and ferritin levels was also reported in iron deficient patients with heart failure [43]. Furthermore, other studies conducted on iron deficient COPD patients also demonstrated a reduction in the walked distance [6,44], reinforcing again this parameter as a hallmark in the overall assessment of severe COPD patients.

Iron homeostasis disruption has been proposed to influence the clinical course of COPD. Specifically, previous studies revealed a relationship between lung function parameters such as FEV_1_ and FVC with different iron metabolism regulators in iron deficient COPD patients [45,46]. In line with these studies, in the present investigation a strong correlation was also found between FEV_1_ and transferrin saturation, suggesting that disruptions in iron metabolism in these patients might exert deleterious effects on their lung function status [45,46]. Further studies are needed to confirm if iron homeostasis may influence lung function and disease severity in COPD.

Hepcidin is a peptide hormone that play an important role in the systemic iron regulation through its interaction with the major iron export protein, the ferroportin [12,14]. In patients with chronic diseases such as chronic hepatitis C, hepatocellular carcinoma, and inflammatory bowel disease, levels of hepcidin were significantly reduced compared to control subjects [47,48,49]. In COPD patients with normal iron metabolism, levels of hepcidin were also reduced [50,51]. In the current study, the iron deficient severe COPD patients were the ones showing a significant decrease in serum hepcidin levels. Furthermore, in the study, hepcidin levels positively correlated with ferritin, suggesting the potential implications of hepcidin in the systemic regulation of iron metabolism. Other studies also showed a positive correlation between those two biological parameters [52,53,54,55,56]. Moreover, serum hepcidin concentration also positively correlated with the six-minute walk distance in the iron deficient group of patients. Collectively, these results put the line forward the potential value of hepcidin as a surrogate marker of iron status and exercise capacity in COPD patients.

Hemojuvelin is a membrane molecule that acts as a co-receptor of bone morphogenetic protein (BMP). Hemojuvelin can negatively regulate hepcidin production through its soluble form [57]. Iron deficient COPD patients exhibited a decline in serum hemojuvelin levels in the current study. To the best of our knowledge, this is the first study that aimed to assess potential differences in serum hemojuvelin levels between iron deficient and normal iron status severe COPD patients. In general, hemojuvelin levels were shown to be increased in anemic patients compared to healthy subjects [58,59]. Among the COPD patients in this study, a significant positive correlation was also found between hemojuvelin and ferritin serum levels, while a negative association was observed between hemojuvelin and the soluble transferrin receptor. Other investigations also demonstrated the existence of a positive correlation between hemojuvelin and ferritin in iron-refractory anemia and in chronic kidney disease patients [59,60,61]. A significant positive correlation between hemojuvelin and hepcidin among all the COPD patients was also observed in the present study. Iron deficient patients exhibited a significant decline in ferritin along with a rise in IRP-1 and IRP-2 levels compared to non-iron deficient COPD patients. Moreover, significant positive correlations were observed between hepcidin and muscle IRP-2 in iron deficient patients. These results reveal the potential crosstalk between muscle and systemic compartment to target iron metabolism regulation. Collectively, the findings reported herein warrant further attention in the study of severe COPD as hemojuvelin and ferritin production may also be influenced by other stimuli such as inflammation [59,62,63], which is a major trigger of organ dysfunction and failure. Future investigations are needed to figure out which parameters can influence the control of the iron-regulatory proteins and to what extent they can affect patients with chronic diseases, in whom the inflammatory component plays a prominent role such as in COPD.

### Study Limitations

Other parameters such as dynamic hyperinflation could have been measured in the study COPD patients. Nonetheless, due to the COVID-19 pandemic, only a few tests were possibly carried out in the lung function testing laboratory of our hospital. The measurements of dynamic hyperinflation were not included as patient recruitment took place to a great extent during the pandemic. It should be mentioned that despite results are shown for both female and male patients, the investigation was not aimed to analyze potential differences between them in each study group. Another potential limitation is related to the cross-sectional design of the study. However, the findings reported herein will help design future intervention studies with a follow-up component.

## 5. Conclusions

In severe iron deficient COPD patients, iron content and its regulators are differentially expressed compared to patients with normal iron content and identical degree of disease severity and muscle weakness. A potential crosstalk between systemic and muscle iron homeostasis regulation was revealed in severe COPD, particularly in the iron deficient patients. Lung function and exercise capacity were directly related to several markers of iron metabolism regulation, thus suggesting a potential role of the iron element in the disease status and severity. Iron regulation status should be included in the overall assessment of COPD patients due to its potential implications on their performance and disease progression.

## Figures and Tables

**Table 1:** General clinical characteristics of the study patients.

	COPD Patients		
	Non-Iron Deficiency	Iron Deficiency	*p* Value
	N = 20	N = 20	
Anthropometry			
Age (years)	68 (8)	66 (8)	0.637
Males/Females	12/8	12/8	1.000
Body weight (kg)	60.3 (11.4)	64.0 (12.7)	0.291
BMI (kg/m^2^)	22.8 (3.5)	24.1 (4.2)	0.287
FFMI (kg/m^2^)	15.7 (2.2)	14.9 (2.2)	0.238
Smoking history			
Active, N (%)	9 (45)	12 (60)	0.525
Ex-smoker, N (%)	11 (55)	8 (40)	
Packs-year	52.5 (32.6)	42.94 (24.8)	0.327
Lung Function			
FEV_1_ (L)	1.19 (0.4)	1.24 (0.4)	0.676
FEV_1_ (% predicted)	46.7 (16.4)	44.0 (11.1)	0.485
FVC (L)	2.8 (0.5)	2.7 (0.8)	0.536
FVC (% predicted)	84.4 (14.5)	76.5 (12.0)	0.071
FEV_1_/FVC	44.8 (11.52)	46.5 (11.1)	0.626
GOLD classification			
1, N (%)	0 (0)	0 (0)	0.224
2, N (%)	9 (45)	5 (25)	
3, N (%)	9 (45)	12 (60)	
4, N (%)	2 (10)	3 (15)	
A, N (%)	11 (55)	9 (45)	0.699
B, N (%)	7 (35)	9 (45)	
C, N (%)	1 (5)	1 (5)	
D, N (%)	1 (5)	1 (5)	

Data are presented as mean (SD). Abbreviations: COPD, chronic obstructive pulmonary disease; BMI, body mass index; FFMI, fat-free mass index; N, number of patients; FEV_1_, forced expiratory volume in one second; FVC, forced vital capacity; GOLD, Global Initiative for Chronic Obstructive Lung Disease. Adjusted *p* values for gender differences are shown in the table.

**Table 2:** Exercise and muscle function assessment of the study patients.

	COPD Patients		
	Non-Iron Deficiency	Iron Deficiency	*p* Value
	N = 20	N = 20	
Six-minute walk test			
Distance (m)	475.74 (63.29)	430.50 (64.77)	0.035
Distance (% predicted)	97.37 (17.58)	84.22 (17.14)	0.019
Upper limb muscle strength			
D-HGS (kg)	25.83 (7.38)	28.42 (8.42)	0.269
D-HGS (% predicted)	87.52 (18.30)	93.19 (20.42)	0.385
ND-HGS (kg)	23.64 (7.51)	25.16 (9.03)	0.648
ND-HGS (% predicted)	89.26 (23.68)	89.79 (21.12)	0.955
Lower limb muscle strength			
D-QMVC (kg)	22.38 (6.60)	22.54 (4.19)	0.332
D-QMVC (% predicted)	62.70 (21.37)	62.29 (12.38)	0.907
ND-QMVC (kg)	21.29 (5.69)	21.69 (4.80)	0.480
ND-QMVC (% predicted)	60.13 (21.54)	60.05 (13.97)	0.953

Data are presented as mean (SD). Abbreviations: COPD, chronic obstructive pulmonary disease; D, dominant; ND, non-dominant; HGS, hand grip strength; QMVC, quadriceps maximum voluntary contraction. Adjusted *p* values for gender differences are shown in the table.

**Table 3:** Iron metabolism parameters in the study patients.

	COPD Patients		
	Non-Iron Deficiency	Iron Deficiency	*p* Value
	N = 20	N = 20	
Iron status			
Hemoglobin (g/dL)	15.2 (1.4)	15.0 (1.6)	0.580
Hematocrit (%)	45.5 (4.7)	44.8 (4.4)	0.613
MCV (fL)	93.7 (3.5)	91.7 (6.7)	0.250
MCH (pg)	31.4 (1.4)	30.7 (2.8)	0.311
MCHC (g/dL)	33.5 (1.3)	33.4 (1.2)	0.820
Ferritin (ng/mL)	214.8 (60.3)	66.5 (33.0)	0.000
Transferrin saturation (%)	31.0 (7.5)	25.4 (7.2)	0.024
Transferrin (g/dL)	236.6 (31.0)	260.7 (28.9)	0.012
Soluble transferrin receptor (mg/L)	2.2 (0.5)	3.0 (0.8)	0.002
Serum iron (µg/dL)	105.8 (26.3)	94.0 (28.8)	0.181
Muscle iron content (mg/g)	0.5 (0.2)	0.3 (0.1)	0.002

Data are presented as mean (SD). Abbreviations: COPD, chronic obstructive pulmonary disease; MCV, mean corpuscular (erythrocyte) volume; MCH, mean corpuscular hemoglobin; MCHC, mean corpuscular hemoglobin concentration. Adjusted *p* values for gender differences are shown in the table.

## Data Availability

The datasets generated and analyzed during the current study are available from the corresponding author on reasonable request.

## Supplementary Materials

The following supporting information can be downloaded at: https://www.mdpi.com/article/10.3390/nu14193929/s1, Figure S1: Representation of scatter plots of correlations between serum ferritin (A), transferrin saturation (B), serum iron (C), serum soluble transferrin receptor, and correlations between serum ferritin (D) and serum iron (E) with transferrin saturation. Representation of scatter plots of correlations between transferrin and soluble transferrin receptor (F), ferritin (G) and transferrin saturation (H).
