# Supplementary material for: Iron Depletion in Systemic and Muscle Compartments Defines a Specific Phenotype of Severe COPD in Female and Male Patients: Implications in Exercise Tolerance
Source: Nutrients. 2022 Sep 22;14(19):3929. doi: 10.3390/nu14193929 (PMC9571884; doi:10.3390/nu14193929)
Supplement: Supplementary file 1 [file nutrients-14-03929-s001.zip › nutrients-1865887-supplementary.pdf]

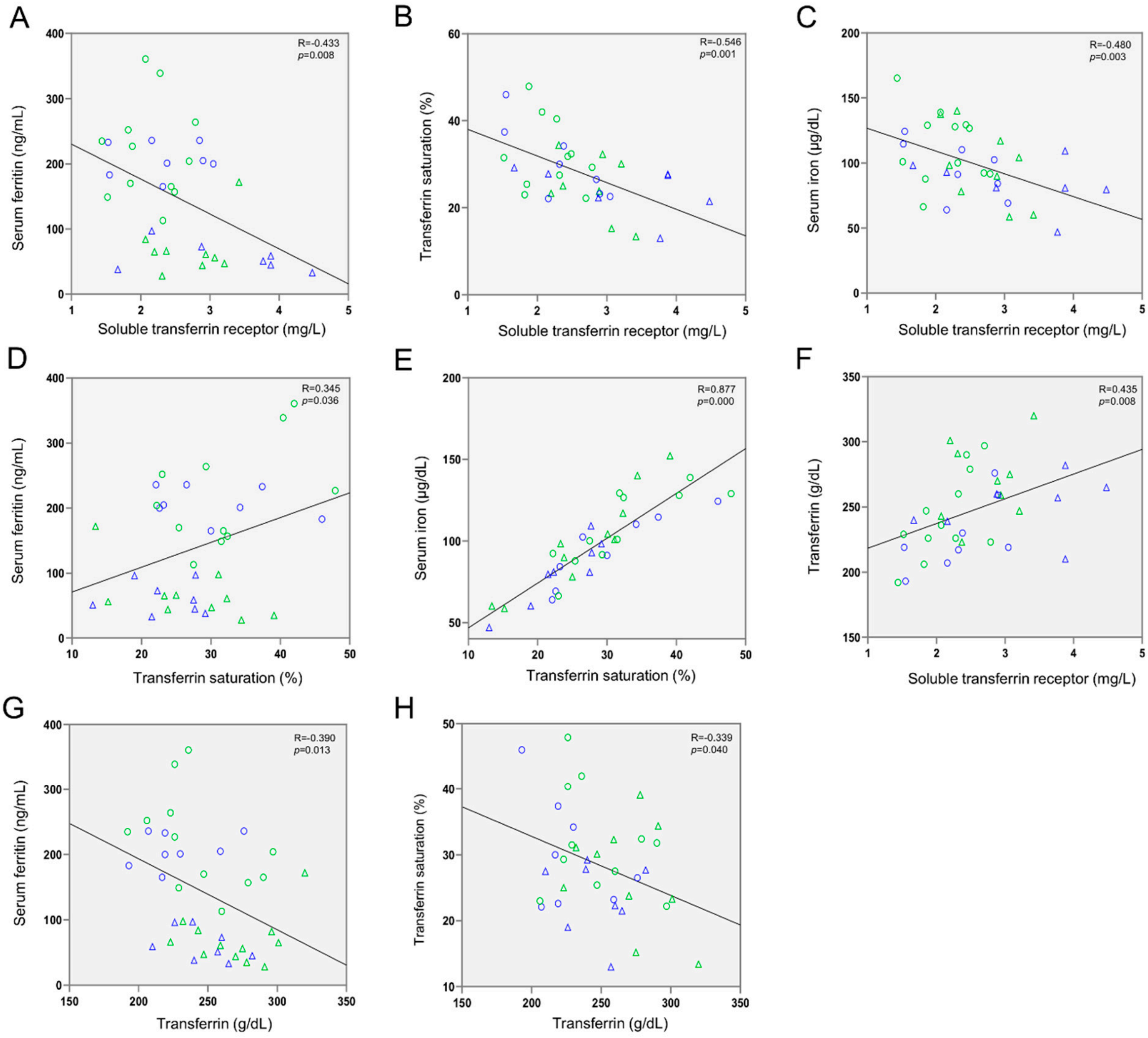

Figure S1. Scatter plot representation of correlations between serum ferritin (A), transferrin saturation (B), and serum iron (C) and serum soluble transferrin receptor, and correlations between serum ferritin (D) and serum iron (E) with transferrin saturation. Scatter plot representation of correlation between transferrin and soluble transferrin receptor (F), ferritin (G) and transferrin saturation (H). In the scatter plot, the green color indicates male patients, while the blue color indicates female patients. The dots represent the patients with non-iron deficiency while the triangles represent the patients with iron deficiency. Abbreviations: COPD, Chronic obstructive pulmonary disease.
